# Study of the physical and chemical changes during the maturation of three cocoa clones, EET8, CCN51, and ICS60

**DOI:** 10.1002/jsfa.9882

**Published:** 2019-07-27

**Authors:** Andrés F Cubillos Bojacá, María C García Muñoz, Ana M Calvo Salamanca, Guillermo H Carvajal Rojas, Martha P Tarazona‐Díaz

**Affiliations:** ^1^ Process Engineering and Industrial Systems Research Group, Department of Engineering Universidad Jorge Tadeo Lozano Bogotá Colombia; ^2^ Corporación Colombiana de Investigación Agropecuaria – Agrosavia Centro de Investigación Tibaitatá Mosquera–Bogotá Colombia

**Keywords:** cacao, variety, maturity, postharvest, raw material, indicator of ripeness

## Abstract

**BACKGROUND:**

Colombia is known for its production of fine and aromatic cocoa; however, the lack of homogeneity in the ripeness stage of cocoa fruit affects the final quality of cocoa beans. Therefore, the aim of this work was to identify parameters that can be use as indicators of ripeness in cocoa fruit in order to homogenize the characteristics of raw cocoa used in the production of cocoa‐products industry. The parameters evaluated were fruit, seed and pod weight, firmness, color, polar and equatorial diameters of the fruit, seed moisture content, total titratable acidity, pH, and total soluble solids of pulp.

**RESULTS:**

Factors such as seed weight, firmness, diameters, total soluble solids, pH, and acidity were affected by the clone factor, whereas seed weight, pH, and total titratable acidity were affected by ripeness stage.

**CONCLUSION:**

Identification of indicators of ripeness for cocoa fruit is a complex task due to the influence of the clone on the evolution of the physicochemical characteristics of cocoa fruit during its maturation process. Thus, indicators must be developed for each clone, and at least two parameters among color, pH, and total titratable acidity should be used to determine the ripeness stage of cocoa fruit. © 2019 The Authors. *Journal of The Science of Food and Agriculture* published by John Wiley & Sons Ltd on behalf of Society of Chemical Industry.

## INTRODUCTION

Cocoa is the main raw material used for the production of cocoa butter, cocoa liquor, chocolate, and other cocoa‐based products. Cocoa products are recognized for their highlighted organoleptic properties, but also for their therapeutic properties regarding health benefits on chronic diseases, like skin care^1^ and cardiovascular diseases.^2^ These properties have boosted cocoa's marketability, and its annual growth is forecast to increase 3.76% annually from 2017 to 2025.^3^ In global terms, Africa contributes about 66.3% of the world cocoa beans production, followed by Asia (17.5%), America (14.9%), and Oceania (1.3%).^4^ Colombia is among the ten leading cocoa‐producing countries worldwide,^4^ with a cocoa area of 175 000 ha and a production of 60 535 tons in 2017; and the Santander region is the biggest contributor, with 39% of the national production.^5^

Colombian cocoa is recognized worldwide as fine cocoa; however, inadequate cultural and postharvest practices lead to low‐quality cocoa for chocolate‐making processes.^6^ Cocoa quality is also affected by other factors, such as environmental and growing conditions, genetic factors, fruit ripeness stage, and pulp preconditioning practices.^7^, ^8^, ^9^ These factors can affect the performance of fermentation and drying processes of cocoa beans, and thus its final quality represented in its aroma profile and taste properties.

In order to homogenize the quality of cocoa beans, innovators have ventured in different aspects, such as the use of stainless steel fermenters to reduce microbial contamination and to have a better control on the inoculum composition,^10^ as well as in analytical methods for the recognition of varieties^11^ with similar characteristics. However, for harvesting, there are no indicators of ripeness that allow the harvesting of cocoa fruit with similar characteristics in order to ensure the homogeneity of the cocoa beans for fermentation and downstream operations. One of the most common harvest parameters used by cocoa farmers is pod color. Nevertheless, this feature also depends on the variety, which ranges from yellow, red to the violet gamut^8^ for most of the mature cocoa fruit. This makes cocoa harvest in a homogeneous ripeness stage a complex and subjective activity. Consequently, the aim of this work was identify objective parameters that can be used as an indicator of ripeness for the three most common clones cultivated in Santander, Colombia. These indicators can be useful for standardizing the raw material and contributing to get a more homogeneous and high‐quality cocoa product.

## Materials and methods

### Plant material

The cocoa clones CCN51, EET8, and ICS60 were collected in the municipality of El Carmen de Chucurí, in the department of Santander (6°41′53″N, 73°30′40″W). Seventy‐five cocoa fruit from these three clones and five ripening stages (25 for each clone, with five fruits for ripeness stage (RS)) were analyzed. The main parameter used for sorting by RS was the pod color, as our previous studies did not show any relation between days after flowering and the ripening process. This time was important only in the growing phase, not along the maturation process (data not shown).

### Preparation of samples

Cocoa fruit harvested and classified according to their ripeness stage RS were transported at 3.0 ± 1.0 °C to the laboratory at the Colombian Corporation of Agricultural Research – Agrosavia and kept at that temperature until analysis. In this study, cocoa beans were consider formed for two main parts: seeds and pulp or mucilage.

### Equatorial and polar diameter of the pod

The equatorial diameter ED was determined using a caliper gauge (vernier digital caliper; Mitutoyo, São Paulo, Brazil) to measure the maximum distance, in a straight line, in the equatorial zone of the pod. Conversely, the polar diameter PD was established as the maximum distance between the two ends of the pod on its vertical axis. Results are expressed in millimeters.

### Pod firmness

This parameter was determined using a texturometer (Chatillon Digital DFIS‐50, Shanghai, China), applying a force with a cylindrical plunger of 3.5 mm in diameter, which dropped at 60 mm min^−1^. Results were expressed as the average of six measurements taken at different points of the pod (upper quarter, lower quarter, equator, both in the valleys and in the ridges) and expressed in newtons.

### Fruit, pod, and seed weight

All fruits were weighed on a balance (Sartorius digital scale; Sartorius, Madrid, Spain) and later cut to extract the seeds. The weight of each fraction (seeds and pod) was recorded in grams.

### Seed moisture content

Ten seeds per each fruit were placed in a Petri dish in an air oven at 100 °C until constant weight, according to AOAC 931.04 method,^12^ with the results expressed as water mass (grams) per 1000 g of sample.

### Pod color

Color parameters were taken at three points in the pod. One located in the equatorial area, and the other two at the upper and lower sections. A color digital colorimeter (Minolta Tlalnepantla, Mexico City, Mexico) was utilized to fix in the CIELAB space the color coordinates L* (luminosity), a* (balance between red and green) and b* (balance between yellow and blue). From these parameters, chroma (saturation, intensity, or purity of the color), hue angle (arctan(b*/a*)),^13^ and color index (1000 × a*/(L*b)) were also determined.^14^

### The pH, titratable acidity, and total soluble solids content of the pulp

Pulp was detached from the seeds and filtered until get 20 mL of clear extract. The pH was measured using a pH/Ion S220 digital potentiometer (Mettler Toledo AG, Schwerzenbach, Switzerland) according to the AOAC 970.21 methodology described for cocoa beans and their products. Acidity was established using the AOAC 942.15 method^12^ and expressed in terms of citric acid (grams per kilogram). Total soluble solids (degrees Brix) were obtained by taking an aliquot of the extract and depositing it in the prism of a PAL‐1 digital refractometer (Atago Co. Ltd, Tokyo, Japan).

### Statistical analysis

The data were subject to an analysis of variance complemented with Tukey's multiple comparison test, using the GLM procedure (*α* ≤ 0.05) found in the SAS software version 9.4 (SAS Institute Inc., Cary, NC, USA). A unidirectional analysis was also carried out in order to obtain more accurate information about the main characteristics for every clone in every ripening stage.

## RESULTS

Table 1 summarizes the results obtained from a global analysis for the influence of the clone without taking into account the ripeness stage, and Table 2 summarizes the results obtained about the influence of ripeness stage without considering the clone. As Table 1 shows, it is possible to identify seed weight, PD, ED, firmness, total soluble solids, pH, and titratable acidity as discriminant variables for the three clones evaluated. Clone ICS60 exhibits the lowest seed weight, ED, and acidity (*P* < 0.05); clone EET8 has the smallest PD; and clone CCN51 has the highest total soluble solids (20.39 °Bx) and the lowest pH (3.420) and firmness (*P* < 0.05). Regarding ripeness stage (Table 2), it is important to point out that pH and acidity showed marked significant differences (*P* < 0.05) among RS5 and the first three RSs (RS1, RS2 and RS3). These results are positive and lead us to consider them as candidate indicators of maturity. Although seed weight also allowed discrimination between RS4 and RS1 its performance was not completely clear, showing a wave behavior during its ripening process.

**Table 1:** Physicochemical variables discriminant for the clones CCN51, EET8, and ICS60

Variable	CCN51	EET8	ICS60
Seed weight (kg)	0.19330 ± 0.0116^ab^	0.19729 ± 0.0234^a^	0.15796 ± 0.0178 ^b^
Total soluble solids (°Bx)	20.39 ± 0.58^a^	16.43 ± 0.68^b^	17.53 ± 0.51^b^
pH	3.420 ± 0.085^b^	3.672 ± 0.143^a^	3.642 ± 0.127^a^
Titratable acidity (g kg^−1^ citric acid)	14.988 ± 1.332^a^	14.678 ± 2.02^a^	12.907 ± 1.877^b^
PD (mm)	211.61 ± 9.82^a^	186.47 ± 9.42^b^	205.53 ± 12.38^a^
ED (mm)	91.82 ± 3.39^ab^	96.44 ± 3.55^a^	90.77 ± 1.92^b^
Firmness (N)	55.33^b^	61.63^a^	59.46^a^

Data are expressed as mean plus/minus standard deviation (*n* = 5). Values in the same line with different superscript letters are significantly different at *P* < 0.05.

**Table 2:** Physicochemical variables discriminant for the ripeness stages

Variable	RS1	RS2	RS3	RS4	RS5
Seed weight (kg)	0.2083 ± 0.0153^a^	0.1733 ± 0.0168^ab^	0.1895 ± 0.0244^ab^	0.1634 ± 0.0149^b^	0.1901 ± 0.0152^ab^
pH	3.536 ± 0.067^b^	3.558 ± 0.0426^b^	3.532 ± 0.055^b^	3.572 ± 0.103^ab^	3.692 ± 0.198^a^
Titratable acidity (g kg^−1^ citric acid)	16.242 ± 0.993^a^	15.129 ± 2.092^ab^	14.500 ± 0.588^ab^	12.914 ± 0.271^bc^	12.181 ± 1.796^c^

Data are expressed as mean plus/minus standard deviation (*n* = 5). Values in the same line with different superscript letters are significantly different at *P* < 0.05.

However, to obtain more specific information on the behavior of each clone during the ripening process a statistical analysis was also performed independently for each one of the clones. The results are consolidated in Table 3 and described in the following.

**Table 3:** Parameters that can be used as discriminant of ripening state for CCN51, EET8 and ICS60 clones

	Physical parameters			Color parameters			Chemical parameters		
	Diameter (mm)								
Clone maturity stage	Equatorial	Polar	Pod firmness (N)	Chrome	Hue angle	Color index	pH pulp	TA (g citric acid kg^−1^ pulp)	TSS pulp (°Bx)
**CCN51**									
1	91.79 ± 3.73^A a^	214.98 ± 14.66^A a^	55.05 ± 1.79^B b^	19.10 ± 3.20^B c^	38.93 ± 9.22^B a^	45.57 ± 13.34^A a^	3.46 ± 0.12^A ab^	15.3 ± 1.5^AB ab^	20.42 ± 1.11^A a^
2	86.71 ± 17.83^A a^	211.89 ± 22.95^A a^	51.76 ± 5.80^BC bc^	26.62 ± 4.62^B b^	39.78 ± 9.37^C a^	46.24 ± 11.85^A a^	3.52 ± 0.14^A a^	17.3 ± 3.3^A a^	20.00 ± 3.00^A a^
3	97.35 ± 5.45^A a^	227.48 ± 12.13^A a^	47.27 ± 5.28^B c^	26.91 ± 3.56^B b^	44.36 ± 8.18^B a^	34.88 ± 8.33^A a^	3.46 ± 0.14^A ab^	14.7 ± 1.4^A ab^	19.94 ± 0.42^A a^
4	91.00 ± 2.90^A a^	205.57 ± 17.08^AB a^	56.92 ± 5.89^AB ab^	37.04 ± 4.41^B a^	39.77 ± 6.45^C a^	38.88 ± 10.11^A a^	3.37 ± 0.06^B ab^	13.2 ± 0.6^A b^	20.08 ± 2.08^A a^
5	92.26 ± 7.21^B a^	198.12 ± 25.68^A a^	62.85 ± 9.80^A a^	42.68 ± 5.42^B a^	43.48 ± 7.20^B a^	34.24 ± 9.27^A a^	3.28 ± 0.06^B b^	14.5 ± 1.3^A ab^	21.50 ± 1.71^A a^
**EET8**									
1	97.01 ± 7.72^A a^	188.90 ± 22.60^A a^	62.75 ± 5.66^A a^	19.37 ± 1.43^B c^	45.16 ± 14.44^B a^	42.11 ± 19.78^A a^	3.63 ± 0.18^A ab^	17.6 ± 3.5^A a^	15.42 ± 1.24^C a^
2	92.70 ± 8.91^A a^	179.42 ± 28.54^A a^	61.13 ± 5.03^A a^	22.04 ± 2.77^B c^	59.08 ± 7.81^B a^	19.62 ± 7.34^B a^	3.53 ± 0.18^A b^	15.8 ± 3.5^AB ab^	16.23 ± 1.94^B a^
3	94.38 ± 9.96^A a^	184.87 ± 29.07^B a^	61.853 ± 2.77^A a^	33.64 ± 3.83^A b^	47.83 ± 8.04^B a^	27.59 ± 10.28^A a^	3.54 ± 0.13^B b^	15.1 ± 2.9^A ab^	16.63 ± 1.84^B a^
4	95.13 ± 7.46^A a^	176.05 ± 39.07^B a^	61.51 ± 4.59^A a^	43.44 ± 3.92^AB a^	53.75 ± 9.68^B a^	20.03 ± 10.59^B a^	3.76 ± 0.19^A ab^	13.0 ± 2.1^A b^	17.53 ± 3.01^A a^
5	102.97 ± 4.90^A a^	203.11 ± 12.90^A a^	60.16 ± 2.57^A a^	44.73 ± 3.09^B a^	50.96 ± 10.44^B a^	23.69 ± 10.35^A a^	3.91 ± 0.17^A a^	11.9 ± 2.5^B b^	16.32 ± 1.28^B a^
**ICS60**									
1	92.07 ± 7.13^A a^	189.78 ± 29.07^A a^	60.35 ± 1.76^A ab^	26.28 ± 4.78^A d^	104.36 ± 2.37^A a^	−5.53 ± 2.00^B d^	3.52 ± 0.06^A b^	15.9 ± 2.8^A a^	17.64 ± 1.49^B a^
2	88.83 ± 4.68^A a^	205.58 ± 15.32^A a^	62.92 ± 3.27^A a^	32.66 ± 4.55^A c^	98.42 ± 3.76^A ab^	−3.91 ± 1.77^C d^	3.61 ± 0.08^A b^	12.3 ± 1.1^B abc^	16.60 ± 0.95^AB a^
3	88.13 ± 6.24^A a^	194.90 ± 11.08^AB a^	62.40 ± 4.11^A a^	33.53 ± 3.30^A c^	97.75 ± 3.96^A b^	−3.46 ± 1.45^B d^	3.59 ± 0.03^AB b^	13.7 ± 1.5^A ab^	17.44 ± 0.82^B a^
4	91.81 ± 8.23^A a^	224.12 ± 33.24^A a^	51.11 ± 2.54^B b^	43.87 ± 4.63^A b^	87.39 ± 5.72^A c^	0.50 ± 1.96^C c^	3.59 ± 0.02^B b^	12.5 ± 2.3^A abc^	17.98 ± 0.25^A a^
5	93.00 ± 7.11^AB a^	213.28 ± 23.78^A a^	60.63 ± 2.90^AB ab^	50.43 ± 6.90^A a^	77.42 ± 7.50^A d^	4.14 ± 2.59^B b^	3.89 ± 0.10^A a^	10.1 ± 0.6^B c^	18.00 ± 1.79^B a^

Data are expressed as mean plus/minus standard deviation (*n* = 5). Mean values within clone followed by different uppercase letters are significantly different at *P* < 0.05. Mean values within the same maturity state followed by different lowercase letters are significantly different at *P* < 0.05.

TA: titratable acidity; TSS: total soluble solids.

### ED and PD of the pods

According to Table 3, it can be stated that neither ED nor PD showed significant differences due to RS; at RS5, clone EET8 showed a larger ED than clone CCN51 did (*P* < 0.05). Regarding PD, the only difference found among clones was at RS4, in which clone EET8 showed the shortest PD (176.05 mm) (*P* < 0.05)

### Pod firmness

**Figure 1:**
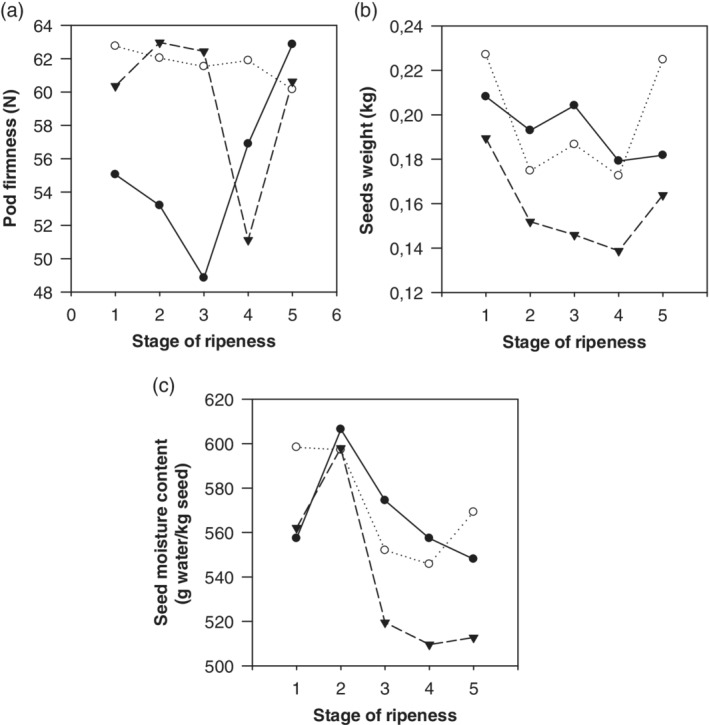
Changes in (a) pod firmness, (b) seeds weight, and (c) seed moisture content of three cocoa clones (CCN51 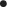, EET8 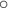, and ICS60 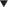) in five stages of ripening.

According to Table 3, the firmness of clone EET8 did not depend on the RS, whereas for clones CCN51 and ICS60 the firmness was affected by RS (*P* < 0.05). Clones showed different behavior in firmness evolution, without a clear trend, as can be observed in Fig. 1(a). However, when the cocoa fruit are fully mature (RS5) these differences disappear and all clones achieve similar firmness values.

### Fruit, pod, and seed weights

The statistical analyses did not show significant differences due to RS for any of the three clones. Fruit, seed, and pod weights were in the ranges 0.630–0.850 kg, 0.139–0.227 kg, and 0.452–0.634 kg respectively. Clones did not reveal significant differences due to RS; Fig. 1(b) illustrates how the seed weight content decreases throughout the ripening process, and also confirms clone ICS60 as recording the lowest seed weight content.

### Seed moisture content

Figure 1(c) shows the behavior of seed moisture content throughout the ripening process of each clone. Seed moisture was not discriminant for RS nor for the clone, although it decreases with ripening. According to some authors,^15^ cocoa bean moisture values for CCN51 and Forastero varieties are 51.89 ± 1.74% and 51.91 ± 0.74% respectively, values similar to those reported in this study for cocoa at RS4 and RS5, stages that corresponds to mature cocoa fruit.

### Color

Table 3 also summarizes the results obtained for the color parameters in each clone throughout the ripening process. Chroma analysis shows that only RS3 allowed differentiation of clone CCN51 from EET8, whereas RS1, RS2, and RS5 allowed differentiation of clone ICS60 from CCN51 and EET8. Hue angle allowed discrimination (*P* < 0.05) between clones CCN51 and EET8 at RS2 and RS4, and among these two clones and clone ICS60 (*P* < 0.05) throughout all five RSs. Color index was different (*P* < 0.05) for CCN51 and EET8 at RS2 and RS4. This means that, despite the similar color that these two clones show, it is possible to differentiate them at RS2 and RS4 by using the color index; at the others RS result difficult identify them. On the other hand, clone ICS60 recorded the lowest color index values for all five maturity stages. Regarding maturity stage, it can be stated that chroma depends on the RS, as chroma values increase as cocoa fruit ripen. This behavior was a constant in all three clones. Hue angle did not discriminate among RSs for clones CCN51 and EET8; however, for clone ICS60 this variable increased with the maturation process. Finally, color index was discriminant for RS only for clone ICS60, increasing along with the ripening process. According to these values, it can be stated that clone CCN51 changes from light pink to dark red and clone EET8 changes from red to orange, whereas clone ICS60 ripens from green to yellow. This agrees with the color description by Vignoni *et al*.^14^ Currently, there are no studies that relate the aforementioned parameters; only Graziani de Fariñas *et al*.^16^ showed that cocoa colors range from purple red, dark red and reddish orange, to a light green with yellow tones depending on the variety, ripeness, and cultivation plot. Color studies are usually carried out for fermented cocoa beans, as this is a quality parameter for commercialization.^17^, ^18^

### pH

**Figure 2:**
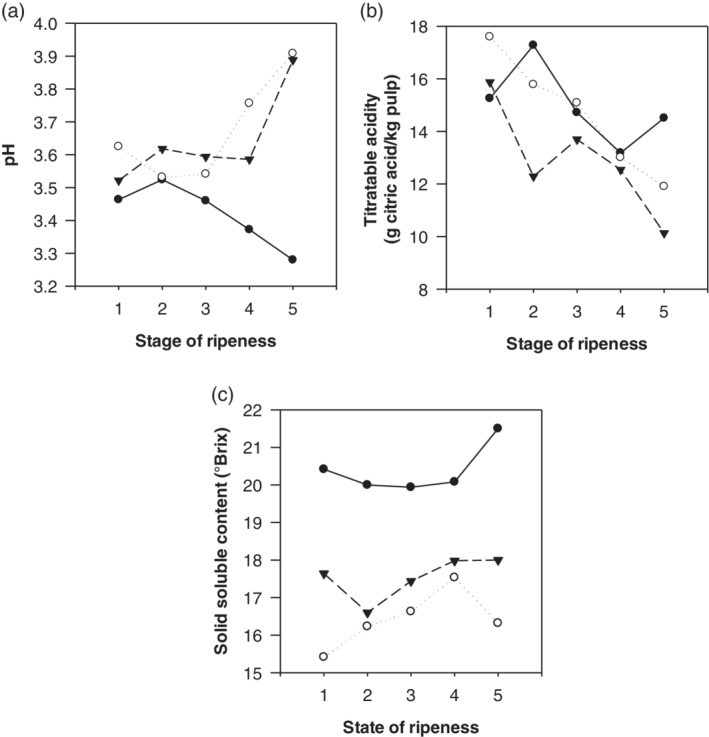
Change in (a) pH, (b) total acidity, and (c) solid soluble content in pulp of three cocoa clones (CCN51 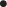, EET8 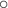, and ICS60 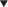) in five stages of ripening.

The pH showed similar values for all three clones at the first ripening stages (RS1 and RS2); differences (*P* < 0.05) were observed only at the advanced RS (RS4, RS5). CCN51 showed a contrasting behavior (Fig. 2(a)). Whereas pH increased for EET8 and ICS60 during the ripening process, for CCN51 this value decreased, achieving the lowest pH values at RS5 (*P* < 0.05). The results obtained in the present study are in the range of those established in other studies: 3.87,^19^ 4.20 ± 0.03,^15^ 3.02–3.40,^20^ and 3.64 ± 0.13 and 3.30 ± 0.88^21^ for different cocoa clones.

### Titratable acidity

The titratable acidity of the cocoa pulp varied between 10 and 18 g of citric acid per kilogram of pulp, depending on the material and the RS (Fig. 2(b)). According to the statistical analysis (Table 3), significant differences (*P* < 0.05) were found among CCN51 and ICS60 at RS2, and between CCN51 and EET8 at RS5. In both cases, clone CCN51 showed the highest acidity value (*P* < 0.05). Titratable acidity was also discriminant for RS, since it decreased with the ripeness process in all three clones.

The data obtained were similar to those reported by Peláez *et al*.,^15^ where the acidity of the cocoa pulp mixed between the Forastero and the CCN51 showed a value of 0.96 ± 0.19% of acetic acid (10.2 ± 2 g of citric acid by kilogram of pulp). According to Prasad *et al*.,^22^ acidity is a more reliable parameter than pH during ripeness establishment, because it is not affected by buffer substances, which are usually found in juices or fruit extracts.

### Total soluble solids

Total soluble solids reached values between 15 and 22 °Bx (Fig. 2(c)), whose variability is explained only by the clone (Table 3), with clone CCN51 reporting the highest value compared with clones EET8 and ICS60 in most of the RSs. The results obtained resemble those published by Vallejo *et al*.,^19^ who found 16 °Bx for the CCN51 clone; also, Álvarez *et al*.^21^ reported values of soluble solids for mature cocoa pulp obtained from the areas of Chuao, Cuyagua, and Cumboto (20.93 ± 0.94 °Bx, 14.89 ± 1.65 °Bx, and 13.24 ± 2.86 °Bx respectively), similar to those found in this study.

## DISCUSSION

The results show how the clone affects physical characteristics like color, pod firmness, and polar diameter, but also important parameters for cocoa quality like pH, acidity, and total soluble solids. Therefore, the identification of indicators of ripeness must be carried out for every clone independently to obtain more reliable data for each indicator. Seed weight content was discriminant for clone independently of RS. This variable determines cocoa yield, which is the reason why clone CCN51, which reported the highest seed weight content, is one of the most cultivated clones in countries like Ecuador and Colombia. Then, it can be said that genetic factors play a crucial role not just in the quality of cocoa products but also in the yield. Concerning the ripeness stage and the effect on the quality of cocoa fruit and cocoa products, some interesting results were found. It was evident that fruit, seed, and pod weights and polar and equatorial diameters are not good parameters for determining ripeness stage, as they did not show significant differences due to RS. This can be explained by the fact that samples were taken after cocoa fruit had reached its physiological maturity stage, after which fruit size and weight no longer change. As with any fruit, cocoa fruit development has three phases after fruit set: growth, maturation, and ripening. In the first one, the fruit increases its size; in the second one it accumulate carbohydrates, such as starch and sucrose, and many other biochemical changes take place until it reaches its physiological mature stage; and after this the ripening process begins.^23^ This means changes in color, starch into sugar, production of volatiles and flavor compounds, softening, changes in proteins, organic acids, amino acids, and lipids, among other changes, but no changes in size or weight. Then, as cocoa fruit were harvested after reaching the physiological phase, the results are totally in line with the normal process of fruit development.

Seed moisture is an important parameter to consider because it has been stated that raw cocoa with high moisture content could lead to cocoa products with high acid content,^9^ which is detrimental for quality of cocoa products.

Another parameter that was considered for this study was pod firmness, but the results showed no very positive results, as its behavior was not as expected. Most fruit, such as cape gooseberry,^24^ mango, papaya, and banana,^25^ show a decrease in firmness during the ripening process, due to cell wall degradation. However, the complex structure of the cocoa pod could have hindered the depolymerization reactions associated with the degradation of the cell wall. Thus, pod firmness is not the best candidate to become an indicator of ripeness for cacao fruit.

The results confirm the fact that color is the most useful parameter to identify the clone, and also its degree of ripeness. Color discriminates perfectly clones that show different color parameters, like ICS60 compared with EET8 or CCN51. But for discriminating among these latter two clones, or among ripeness stages, only the chroma parameter can be recommended. It is difficult to find only one parameter that can differentiate among clones but also ripeness stage in each clone, considering that there are so many types of cocoa clones, and they express their ripeness in different ways, depending on the types of pigments they contain. This means that both genetic factors and ripeness stage determine the color of cocoa fruit. Consequently, although chroma can be recommended as a potential indicator of ripeness, it should be used in combination with other parameters to get more reliable information about ripeness stage in view of the fact that this parameter does not discriminate for every ripeness stage.

The pH could be a parameter to use as an indicator of ripeness, but it should be combined with other parameters, like color, owing to the fact that it showed significant differences in all three clones but not for every one of the RSs, except for extreme ripeness stages such as between RS1 and RS4. This is useful, as raw cocoa at RS1 is not able to develop a complete flavor profile throughout its processing along with the fermentation and downstream operations. However, it is recommended to identify ripeness indicators that allow one to differentiate among closer RSs.

Concerning the acidity, similar results to those for pH were found. However, this parameter was able to differentiate among closer ripeness stages which allows the harvest of more homogeneous cocoa fruit. Clone ICS60 had the lowest acidity, which is beneficial for quality of cocoa products and also permits use of cocoa clones with high titratable acidity that can be mixed with this ICS60 clone to get a more appropriate raw cocoa for processing.

Total soluble solids, along with the color, is the most common parameter used as an indicator of ripeness in fruit; however, this was not the case for cocoa. The ample variability in the sugar content of cocoa at different ripeness stages impedes its use as an indicator of ripeness. Total soluble solids also depends on genetic factors, which was evident and confirmed in this study; but it also depends on the environmental conditions, soil composition, water availability, and other cultural practices that can make that conversion of starch into sugar fast or slow. This study was carried out in the same region and farms to reduce the effect of those other factors. However, the position of the cocoa fruit in the tree was not taken into account, and this could affect the velocity of the accumulation of sugars in cocoa fruit. Fruit with longer solar light exposure could enhance the advance of the sugar accumulation. It could be that these additional factors hindered the effect of the clone or of the RS on the total soluble solids accumulation. In consequence, the results found for this parameter do not allow us to propose it as an indicator of ripeness.

These differences in physicochemical characteristics in the raw material are responsible for low quality in the final cocoa beans. Therefore, the previous classification of the fruits looking for more homogeneous characteristics must be an irrefutable activity to contribute to the final quality of cocoa products.

Seed moisture content, color (chroma and hue angle), titratable acidity, and pH were also affected by the ripening stage. Most of these effects were more evident in ICS60 than in the other two clones. Taking into account the crucial importance of the homogeneous raw cocoa in the final quality of cocoa products, it is essential to continue working to identify a more accurate indicator of ripeness for cocoa fruit.

Finally, pH is an important parameter to take into account for raw cocoa, taking into account that this parameter determines the development of microbial communities and the activity of key enzymes during fermentation.

## CONCLUSIONS

The results show that identifying indicators of ripeness for cocoa fruit is a complex task as this fruit does not present the typical behavior showed by most fruit through maturation. Significant differences were found in some of most common indicator of ripeness in fruit but not for every ripeness stage, but only at the extremes of the ripening stage, such as RS1 and RS5 or in the best of the cases among RS1 and RS4. This leads us to suggest the use of combined parameters such as color, pH, and acidity to get a more reliable and complete information about the degree of ripeness of cocoa fruit. Harvesting cocoa fruit with similar characteristics should contribute to a more homogeneous raw cocoa for processing.

It is recommended to continue with these kinds of studies until we get more reliable and also practical indicators of ripeness that can be used by farmers and then to get a better raw material for the chocolate industry. Carrying out deeper studies that include changes in physical and chemical characteristics of cocoa fruit through the ripening process will allow us to gain insights into how to achieve cocoa products with outstanding sensorial, nutritional, and functional properties.

## CONFLICT OF INTEREST STATEMENT

The authors declare that they have no conflicts of interest.

## References

[1] Scapagnini G , Davinelli S , Di Renzo L , De Lorenzo A , Olarte HH , Micali G *et al*, Cocoa bioactive compounds: significance and potential for the maintenance of skin health. Nutrients 6:3202–3213 (2014). 10.3390/nu6083202. 25116848 PMC4145303

[2] Buitrago‐Lopez A , Sanderson J , Johnson L , Warnakula S , Wood A , Di Angelantonio E *et al*, Chocolate consumption and cardiometabolic disorders: systematic review and meta‐analysis. Br Med J 343:d4488 (2011). 10.1136/bmj.d4488. 21875885 PMC3163382

[3] Pacyniak B , $9.9‐billion cocoa market to grow 3.76 percent annually. Candy Ind 183:11–12 (2018).

[4] Food and Agriculture Organization of the United Nations , FAOSTAT, food and agriculture data [Online]. FAO, Rome, Italy (2018) Available: http://www.fao.org/faostat/en/#home [27 July 2018].

[5] FEDECACAO , National economy. [Online]. Federacion Nacional de Cacaoteros, Bogota, Colombia (2018). Available: https://www.fedecacao.com.co/portal/index.php/es/2015‐02‐12‐17‐20‐59/nacionales [27 July 2018].

[6] Sánchez Vargas AP , Castellanos Domínguez OF and Domínguez Martínez KP , Improvement of the post‐harvest of cocoa from roadmapping. Ing Invest 28:150–158 (2008) (in Spanish, with English abstract).

[7] Caligiani A , Marseglia A , Prandi B , Palla G and Sforza S , Influence of fermentation level and geographical origin on cocoa bean oligopeptide pattern. Food Chem 211:431–439 (2016). 10.1016/j.foodchem.2016.05.072. 27283652

[8] Gutiérrez TJ , State‐of‐the‐art chocolate manufacture: a review. Compr Rev Food Sci Food Saf 16:1313–1344 (2017). 10.1111/1541-4337.12301. 33371587

[9] Afoakwa EO , Kongor JE , Takrama JF and Budu AS , Changes in acidification, sugars and mineral composition of cocoa pulp during fermentation of pulp pre‐conditioned cocoa (*Theobroma cacao*) beans. Int Food Res J 20:1215–1222 (2013).

[10] de Melo Pereira GV , Magalhães KT , de Almeida EG , da Silva Coelho I and Schwan FR , Spontaneous cocoa bean fermentation carried out in a novel‐design stainless steel tank: influence on the dynamics of microbial populations and physical–chemical properties. Int J Food Microbiol 161:121–133 (2013). 10.1016/j.ijfoodmicro.2012.11.018. 23279821

[11] Vargas JP , Ciobotă V , Salinas W , Kampe B , Aponte MP , Rösch P *et al*, Distinction of Ecuadorian varieties of fermented cocoa beans using Raman spectroscopy. Food Chem 211:274–280 (2016). 10.1016/j.foodchem.2016.05.017. 27283632

[12] AOAC , Official Methods of Analysis of AOAC International, 20th edn. AOAC International, Gaithersburg, MD (2016).

[13] Sahin S and Summu S , Electromagnetic properties, in Physical Properties of Foods, ed. by Sahin S and Summu S Springer Science & Business, Ankara, Turkey, pp. 190–203 (2006).

[14] Vignoni LA , Césari RM , Forte M and Mirábile ML , Determination of color index in minced garlic. Inf Tecnol 17:63–67 (2006).(in Spanish, with English abstract)). 10.4067/S0718-07642006000600011.

[15] Peláez PP , Guerra S and Contreras D , Changes in physical and chemical characteristics of fermented cocoa (*Theobroma cacao*) beans with manual and semi‐mechanized transfer, between fermentation boxes. Sci Agropecu 7:111–119 (2016).

[16] Graziani d FL , Ortiz d BL , Angulo J and Parra P , Physical characteristics of Criollo, Forastero and Trinitario cocoa fruit of the town of Cutombo, Venezuela. Agron Trop (Maracay, Venez) 52:343–362 (2002) (in Spanish, with English abstract).

[17] Nogales J , Graziani d FL and Ortiz d BL , Physical and chemical changes during sun drying of the fermented cocoa bean in two designs of wooden crates. Agron Trop 56:5–20 (2006) (in Spanish, with English abstract).

[18] Ortiz d BL , Graziani d FL and Rovedas LG , Influence of several factors on characteristics of fermented and sun‐dried cocoa beans. Agron Trop 59:119–127 (2009) (in Spanish, with English abstract).

[19] Vallejo TCA , Díaz OR , Morales RW , Soria VR , Vera CJF and Baren C , Use of cocoa mucilage, national and trinitarian type, in obtaining jelly. Revista ESPAMCIENCIA 7:51–58 (2010) (in Spanish, with English abstract).

[20] Romero C and Zambrano A , Analysis of sugars in cocoa pulp by colorimetry and capillary electrophoresis. Revista Científica UDO Agrícola 12:906–913 (2012) (in Spanish, with English abstract).

[21] Álvarez C , Pérez E and Lares M , Morphology of the fruits and physical–chemical characteristics of the cocoa mucilage from three zones of Aragua state. Agron Trop 52:497–506 (2002) (in Spanish, with English abstract).

[22] Prasad K , Jacob S and Siddiqui MW , Fruit maturity, harvesting, and quality standards, in Preharvest Modulation of Postharvest Fruit and Vegetable Quality, ed. by Siddiqui MW Academic Press, London, pp. 41–69 (2018).

[23] Seymour GB, Taylor JE and Tucker GA eds, Biochemistry of Fruit Ripening. Chapman & Hall, London (1993).

[24] Ciro VHJ , Buitrago GOH and Pérez ASA , Preliminary study of mechanical resistance to fracture and firmness of uchuva (*Physalis peruviana* L.) fruits. Rev Fac Nac Agron Medellín 60:3785–3796 (2007) (in Spanish, with English abstract).

[25] Torres R , Montes EJ , Pérez AO and Andrade RD , Influence of color and ripeness stages on the texture of tropical fruits (mango, papaya and plantain). Inf Tecnol 26:47–52 (2015). (in Spanish, with English abstract)). 10.4067/S0718-07642015000300008.

